# Different endoscopic treatments for small colorectal polyps: A systematic review, pair-wise, and network meta-analysis

**DOI:** 10.3389/fmed.2023.1154411

**Published:** 2023-04-06

**Authors:** Xuanhan Li, He Zhu, Fudong Li, Ri Li, Hong Xu

**Affiliations:** ^1^Department of Gastroenterology, The First Hospital of Jilin University, Changchun, Jilin, China; ^2^Department of Library, The First Hospital of Jilin University, Changchun, Jilin, China

**Keywords:** colonic polyps, cold snare polypectomy, hot snare polypectomy, therapeutics, meta-analysis, treatment outcome

## Abstract

**Background and study aims:**

In recent years, cold snare polypectomy (CSP) has been increasingly used for small polyps (<10 mm) instead of hot snare polypectomy (HSP). However, evidence-based research regarding the effectiveness and safety of CSP and HSP are still lacking. Additionally, for 4–10 -mm non-pedunculated polyps, the polyp removal method is still controversial. Therefore, it is clinically significant to conduct pair-wise and network meta-analyses to assess such resection methods.

**Methods:**

We searched PubMed, Embase, and the Cochrane library for randomized controlled trials (RCTs). Only studies that involved the resection of polyps <10 mm were included. Outcomes included the complete resection rate, polyp retrieval rate, procedure-related complications, and procedure times.

**Results:**

Overall, 23 RCTs (5,352 patients) were identified. In meta-analysis compared CSP versus HSP for polyps <10 mm, CSP showed lower complete resection rate than HSP although with no statistically significant difference [odds ratio (OR): 0.77, 95% confidence interval (CI): 0.56–1.06]. CSP showed a lower risk of major post-polypectomy complications compared to HSP (OR: 0.28, 95% CI: 0.11–0.73). In the network meta-analysis for 4–10 mm non-pedunculated polyps, HSP, and endoscopic mucosal resection (EMR) showed a higher complete resection rate than CSP (OR: 2.7, 95% CI: 1.3–9.2 vs. OR: 2.6, 95% CI: 1.0–10) but a significantly longer time than CSP (WMD: 16.55 s, 95% CI [7.48 s, 25.25 s], *p* < 0.001), (WMD: 48.00 s, 95% CI [16.54 s, 79.46 s], *p* = 0.003). Underwater CSP ranked third for complete resection with no complications.

**Conclusion:**

For <10 mm polyps, CSP is safer than HSP, especially for patients taking antithrombotic drugs. For 4–10 mm non-pedunculated polyps, HSP, and EMR have higher complete resection rates than CSP.

**Systematic review registration:**

https://www.crd.york.ac.uk/PROSPERO/, identifier CRD42022315575.

## Introduction

Colorectal cancer (CRC) is the third most commonly diagnosed cancer and ranks second in terms of mortality worldwide (1). One of the most effective methods to prevent colorectal cancer is colonoscopy, (2) but the incidence of interval colorectal cancer (cancers found in patients after a screening or surveillance colonoscopy) is 5–17 cases per 10,000 person-years of follow-up (3–5). Interval cancers are mainly caused by missed lesions, incomplete polyp resections, and new cancers. Incomplete polyp resection has been estimated to cause 10–28% of all interval CRCs, (4–7) and should be given the same attention as adenoma detection rates (8). For small polyps (<10 mm), the incomplete resection rate ranges from 6.8 to 15.9% (9–11). Despite this, polypectomy is not without complications. The risk of post-polypectomy bleeding (PPB) ranges from 0.3 to 10% depending on a variety of factors, including the polyp size, location, morphology, and resection technique (12).

For the resection of polyps <10 mm, hot snare polypectomy (HSP) has been used in the past. In recent years, cold snare polypectomy (CSP) has been used more frequently. Studies do not support the superiority of CSP over HSP in complete resection rate (13, 14). However, some studies have indicated that CSP appears to be safer than HSP, especially for patients taking antithrombotic agents (15, 16). These conclusions are limited to single clinical studies, No evidence-based medical research has yet emerged.

For resecting polyps smaller than 5 mm, CSP is accepted in the guidelines (17, 18). For polyps of 4–10 mm, CSP was recommended as it induce less injury to the submucosal arteries than polypectomy methods using electrocautery (19). However, The incomplete resection rate can reach 18.4% in a recent study using CSP, (20) which is worse than a previous study (CARE study) with comparable design using HSP (11). Evidence comparing efficacy with HSP is lacking. Meanwhile, many new evidences and new methods have appeared in recent years. Therefore, there is an urgent need to conduct a widespread and systematic evaluation of the efficacy and safety of different polyp resection treatments, including CSP, HSP, endoscopic mucosal resection (EMR), and new techniques for 4–10 mm non-pedunculated polyps, in order to provide medical evidence for guideline development and clinical practice.

In this study, we aimed to compare the complete resection rates and complication rates between HSP and CSP for polyps smaller than 10 mm. Further, we assessed the efficacy and safety of different methods for 4–10 mm non-pedunculated polyps through direct and indirect comparisons using network meta-analysis.

## Methods

This systematic review and network meta-analysis (NMA) is registered (CRD42022315575) on the International Prospective Register of Systematic Review (PROSPERO). We followed the PRISMA NMA checklist statement for network meta-analysis (Supplementary Table 1).

### Search strategy

We searched PubMed, Embase, and the Cochrane library from inception until August 14, 2022. The Clinical Trial Registry was searched for unpublished trials. Our search strategy is described fully in Supplementary Appendix 1.

### Inclusion and exclusion criteria

All studies included in this meta-analysis were RCTs published in English as full-text articles. Because of the different study populations and interventions, our study used the following two different inclusion criteria: (1) Patients: ① colorectal polyps in adults upon examination were found to be less than 10 mm ② non-pedunculated polyps range from 4 to 10 mm; (2) Intervention: ① CSP, HSP. ② endoscopic treatment for small colorectal polyps, including CSP, HSP, EMR, argon plasma coagulation (APC), underwater cold snare polypectomy (UCSP), cold snare endoscopic mucosal resection (CS-EMR), or underwater endoscopic mucosal resection (UEMR); (3) Comparators: ① CSP vs. HSP ② compared with each other; (4) Outcomes: the primary outcome was the complete resection rate; additional outcomes included procedure-related complications, polyps retrieval rate, and procedure times. We excluded articles that described endoscopic treatment only for <5 mm polyps.

### Data extraction and processing

Independent investigators (Li XH and Zhu H) screened the full texts for eligibility using all inclusion criteria and extracted the study data, with discrepancies adjudicated by Xu H. We extracted first author, publication of year, study region, country, multicenter/non-multicenter experiment, number of patients, characteristics of patients, number of polyps, method of polyp removal, characteristics of polyps, proportion of adenoma, whether the resection was extended using cold snare, and primary and second outcomes, including complete resection rate, polyp retrieval rate, IB rate, PPB rate, major PPB rate, and resection time. Three methods were used to evaluate complete resection rate: negative biopsy, R0 resection rate, and recurrence rate. Extended resection was defined as a polyp resected with normal tissue of >1 mm from the margin using cold snare. IB was defined as bleeding that occurred during colonoscopy after the polypectomy that did not stop spontaneously within 30 s and required any form of endoscopic hemostasis. PPB was defined as hematochezia occurring within 30 days after polypectomy. Major PPB was defined as PPB requiring endoscopic hemostasis or a significant decrease in hemoglobin (1 mg/dL or more) within 30 days after polypectomy. We also contacted the studies’ authors and read related meta-analysis to supplement the incomplete reports of two original papers (15, 21).

### Risk of bias and quality assessment

Two investigators (Li XH and Li R) assessed the studies’ risks of bias in accordance with the Cochrane Handbook for Systematic Reviews of Intervention. We assessed the risk of bias for five outcomes (complete resection rate, polyp retrieval rate, IB rate, PPB rate, and operation time). Furthermore, the GRADE (Grading of Recommendations Assessment, Development, and Evaluation) assessment was used to assess the quality of pair-wise and network estimates based on five aspects: risk of bias, imprecision, inconsistency, indirectness, and publication bias (22).

### Data synthesis and analysis

#### Pair-wise meta-analysis

Standard pair-wise meta-analysis was conducted using a random-effects model. Continuous variables were analyzed by weighted mean difference (WMD) with 95% confidence interval (CI), and dichotomous variables by odds ratio (OR) with 95% CI. The *I*^2^-statistic was calculated to assess the heterogeneity. Additionally, subgroup analysis was used to compare the complications for patients whether on antithrombotic drugs. Pair-wise meta-analysis and subgroup analysis was conducted using RevMan 5 statistical software (version 5.4).

#### Network meta-analysis and sensitivity analysis

A random-effects NMA based on a Bayesian framework was performed through the “gemtc” package in the statistical software R (version 4.1.3). To calculate the relative ranking of interventions for achieving primary and secondary outcomes, we calculated their surface under the cumulative ranking (SUCRA) curve using software R (version 4.1.3). SUCRA reports the overall probability, based on the ranking of all interventions that a given intervention is among the best treatments (23). SUCRA values ranged from 0 (treatment is the worst) to 1 (treatment is the best) (24).

Sensitivity analysis was performed to assess the robustness of the primary outcome of our network meta-analysis. We performed sensitivity analysis after excluding studies that using R0 resection rate to evaluate complete resection rate.

#### Consistency, transitivity, and heterogeneity in network meta-analysis

Our study used a node-splitting model to estimate consistency (the agreement between direct and indirect comparison in NMA). When *P* > 0.05, we consider the results of direct and indirect comparisons to be consistent. Global and local statistical heterogeneity among the studies were assessed by *I*^2^ statistics. We considered an *I*^2^ > 50% to show significant statistical heterogeneity. The transitivity assumption of NMA was evaluated by comparing different variables such as patients and polyp characteristics in different RCTs.

## Results

### Study characteristics

**FIGURE 1:**
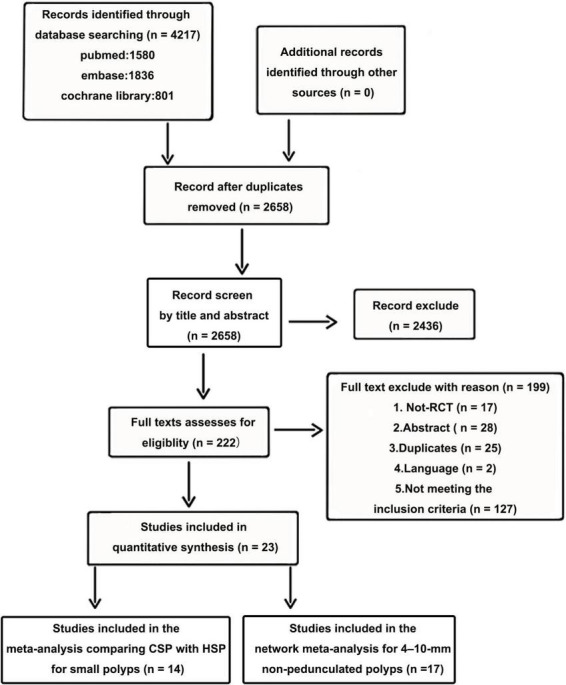
Flowchart of included studies.

**FIGURE 2:**
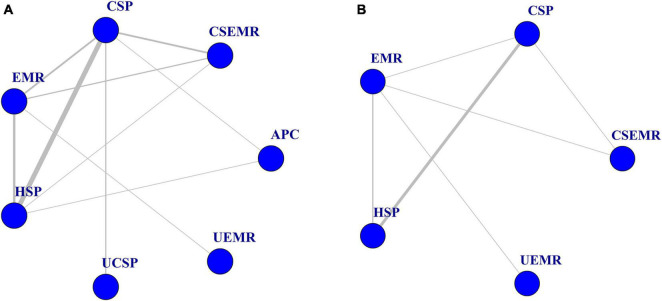
Evidence network of eligible comparisons for network meta-analysis. **(A)** Complete resection rate (*n* = 17). **(B)** Intraprocedural bleeding rate (*n* = 11). Lines connect the interventions that have been compared directly (head-to-head) in the eligible randomized controlled trials (RCTs).

Twenty-three RCTs, including multi-arm studies (5,352 patients) were included in the final quantitative synthesis. A flow chart of the trial selection process is shown in Figure 1. Fourteen articles compared HSP with CSP, (15, 16, 21, 25–35) three articles compared HSP to EMR, (31, 36, 37) three articles compared CSP to CS-EMR, (31, 38, 39) two articles compared CSP to EMR, (31, 40) two articles compared CSP to UCSP, (41, 42) one article compared CS-EMR to EMR, (43) one article compared UEMR to EMR, (44) and one article compared APC to HSP and CSP (33). All studies were published after 2010, and nine studies were multicenter studies. Nine RCTs were conducted in Japan. Fourteen articles were included in the meta-analysis comparing CSP with HSP for small polyps, (15, 16, 21, 25–35). Seventeen articles were included in the network meta-analysis for 4–10 mm non-pedunculated polyps (27–29, 31–44). The characteristics of the included studies are summarized in Table 1. NMA was not performed for outcomes with inadequate studies or low positive events. Figures 2A, B show the evidence network.

**TABLE 1:** Characteristics of included randomized controlled trials.

References	Country	Study period	Inclusion criteria of polyp size	Multicenter	Method	No. of patients	Patients on antithrombotic agent
Aizawa et al. (25)| |	Japan	2013.9–2016.6	≤9 mm	YES	A CSP	139	NO
					B HSP	134	
de Benito Sanz et al. (26)| |	Spain	2017.10–2019.2	5–9 mm	YES	A CSP	232	PART
					B HSP	256	
Horiuchi et al. (15)| |	Japan	2012.3–2012.12	≤10 mm	NO	A CSP	35	ALL
					B HSP	35	
Pedersen et al. (27)| |¶	Europe	2015.8–2020.1	4–9 mm	YES	A CSP*	218	NO
					B HSP	207	
Ichise et al. (21)| |	Japan	2008.9–2009.3	<8 mm	NO	A CSP	40	NA
					B HSP	40	
Ito et al. (28)| |¶	Japan	2015.5–2018.5	6–9 mm	NO	A CSP	59	PART
					B HSP	60	
Kawamura et al. (29)| |¶	Japan	NA	4–9 mm	YES	A CSP	NA	NO
					B HSP	NA	
					C EMR	NA	
Kim et al. (36)¶	Korea	2014.6–2015.12	5–9 mm	NO	A HSP	134	NO
					B EMR	135	
Kim et al. (37)¶	Korea	2014.6–2017.12	5–10 mm	NO	A HSP	142	NA
					B EMR	130	
Myung et al. (42)¶	Korea	2019.12–2020.6	4–9 mm	NO	A CSP*	54	NO
					B UCSP*	56	
Papastergiou et al. (43)¶	Greece	2016.1–2016.10	6–10 mm	YES	A CS–EMR*	77	NO
					B EMR	78	
Paspatis et al. (30)| |	Greece	2010.5–2010.11	3–8 mm	NO	A CSP	208	NO
					B HSP, EMR	206	
Rex et al. (31)| |¶	America	2018.10–2021.3	6–15 mm	YES	A CSP	NA	NO
					B CS–EMR	NA	
					C HSP	NA	
					D EMR	NA	
Shimodate et al. (38)¶	Japan	NA	3–10 mm	NO	A CSP	107	PART
					B CS–EMR	107	
Suzuki et al. (32)| |¶	Japan	2015.7–2017.3	4–10 mm	NO	A CSP*	25	NO
					B HSP	27	
Varytimiadis et al. (33)| |¶	Greece	2015.1–2018.1	5–9 mm	NO	A HSP	NA	NO
					B CSP*	NA	
					C APC	NA	
Yen et al. (41)¶	America	2016.10–2018.9	≥ 6 mm	NO	A UCSP	NA	PART
					B CSP	NA	
Zhang et al. (40)¶	China	2014.3–2016.5	6–9 mm	NO	A CSP*	179	NO
					B EMR	179	
Zhang et al. (44)¶	China	NA	4–9 mm	YES	A EMR	64	NO
					B UEMR*	66	
Takeuchi et al. (16)| |	Japan	2016.6–2017.12	<10 mm	YES	A HSP, EMR	83	ALL
					B CSP	85	
Wei et al. (39)¶	America	2020.9–2021.5	4–9 mm	NO	A CSP	109	NO
					B CS–EMR	105	
Koyanagi et al. (35)¶	Japan	2018.11–2020.7	6–10 mm	YES	A CSP	27	NO
					B HSP	22	
Fatima et al. (34)¶	America	2009.9–2013.5	4–6 mm	NO	A CSP	87	NO
					B HSP	82	
**References**	**Age mean ± SD, median (IQR)**	**Gender female (%)**	**No. of polyps**	**Polyp size, mm mean ± SD, median (IQR)**	**Adenoma (%)**	**Complete resection (n/N)**	**Evaluation of complete resection**
Aizawa et al. (25)| |	65.7 ± 8.8	33.1	369	5.1 ± 1.7	84.7	NA	NA
	66.7 ± 8.8	29.1	360	5.2 ± 1.9	86.4	NA	
de Benito Sanz et al. (26)| |	64.7 (56.7-70.5)	35.3	387	6(5–7)	85.1	358/387	Negative biopsy†
	64.5 (57–70.7)	34	385	6(5–7)	85.6	362/385	
Horiuchi et al. (15)| |	67 ± 13	29	78	6.5 ± 1.2	92.3	65/73	Negative margin‡
	67.3 ± 12	31	81	6.8 ± 1.3	91.3	67/75	
Pedersen et al. (27)| | ¶	63.1 (42–83)	37.2	318	NA	70.1	284/318	Negative biopsy†
	61.9 (40–82)	41.6	283	NA	75.6	262/283	
Ichise et al. (21)| |	65.1 ± 11	38	101	5.7 ± 4.0	94.4	87/97	Negative margin‡
	65.5 ± 12	30	104	5.5 ± 6.0	90.3	85/100	
Ito et al. (28)| |¶	66.8 ± 12.4	35	175	NA	94.8	78/80	No recurrence§
	66.9 ± 9.8	33	157	NA	94.9	79/79
Kawamura et al. (29)| | ¶	NA	NA	341	5.4 ± 1.4	100	335/341	Negative biopsy†
	NA	NA	194	5.4 ± 1.4	100	190/194	
	NA	NA	152	5.4 ± 1.4	100	147/152	
Kim et al. (36)¶	64 ± 10	35.1	172	6.2 ± 1.3	91.9	152/172	Negative biopsy†
	64.3 ± 10.1	42.2	181	6.3 ± 1.4	91.7	168/181	
Kim et al. (37)¶	62.8 ± 10.7	31.7	167	7.1 ± 1.5	100	161/167	Negative biopsy†
	63.0 ± 10.8	36.8	155	7.2 ± 1.6	100	148/155	
Myung et al. (42)¶	58 (35–90)	35	100	5.6 ± 1.5	80.0	59/100	Negative margin‡
	58 (29–87)	23	98	5.9 ± 1.7	79.6	83/98	
Papastergiou et al. (43)¶	63.6 ± 10.6	41.3	83	8.2 ± 1.6	91.6	77/83	Negative biopsy†
	63.1 ± 10.3	40.3	81	8.3 ± 1.4	90.1	78/81	
Paspatis et al. (30)| |	59.4 ± 13.6	49	530	5.3 ± 1.4	83.7	NA	NA
	61.3 ± 11	65	553	5.67 ± 1.3	77.7	NA	
Rex et al. (31)| |¶	NA	NA	41	NA	NA	41/41	Negative biopsy†
	NA	NA	47	NA	NA	47/47	
	NA	NA	36	NA	NA	36/36	
	NA	NA	33	NA	NA	33/33	
Shimodate et al. (38)¶	65	43	100	5	97	58/100	Negative margin‡
	68	43.9	97	5	98	41/97	
Suzuki et al. (32)| |¶	66.9 ± 7.7	24	25	5.8 ± 1.7	88	17/22	Negative margin‡
	66.5 ± 9.8	25.9	27	5.6 ± 1.8	91.3	24/26	
Varytimiadis et al. (33)| |¶	NA	NA	45	7.3 ± 0.9	82.2	44/45	No recurrence§
	NA	NA	39	6.4 ± 0.9	76.9	36/39	
	NA	NA	37	6.4 ± 0.6	NA	35/37	
Yen et al. (41)¶	NA	NA	180	NA	NA	177/180	Negative biopsy†
	NA	NA	164	NA	NA	160/164	
Zhang et al. (40)¶	64.5 ± 7.7	46.6	267	7.4 ± 1.2	80.1	194/212	Negative biopsy†
	65.8 ± 9.4	43.6	258	7.7 ± 1.5	77.9	200/203	
Zhang et al. (44)¶	57.6 ± 9.8	45.3	71	5.0 (4–7)	67.6	62/71	Negative biopsy†
	55.1 ± 11.2	39.4	71	6.0 (5–8)	71.8	59/71	
Takeuchi et al. (16)| |	73 (68–76)	18	286	5.0 (3–6)	86.0	NA	NA
	73 (70–76)	11	325	5.0 (3–6)	93.0	NA	
Wei et al. (39)¶	68.7 ± 7.8	1.2	149	5.3 ± 1.5	84.6	145/149	Negative biopsy†
	68.9 ± 7.9	2.9	142	5.3 ± 1.5	87.3	140/142	
Koyanagi et al. (35)¶	62(40–79)	13.6	35	6.0	80.0	33/35	Negative margin‡
	68(44–79)	37.0	26	6.0	80.8	21/26	
Fatima et al. (34)¶	57.7 ± 6.7	49.4	111	4.8 ± 0.3	97.2	47/52	No recurrence§
	56.7 ± 6.4	53.7	103	4.7 ± 0.4	98.0	50/50	

HSP, hot snare polypectomy; CSP, cold snare polypectomy; APC, argon plasma coagulation; UCSP, underwater cold snare polypectomy; CS-EMR, cold snare endoscopic mucosal resection; UEMR, underwater endoscopic mucosal resection; NA, not applicable.

*Study reported that Polyp resected with a more than 1 mm circumferential margin using cold snare.

†Biopsy specimen obtained from the margin of the polypectomy site.

‡Negative lateral and vertical margins for neoplasia (R0 resection).

§No recurrence in resection site in following surveillance colonoscopy.

| |Articles included in the meta-analysis comparing CSP with HSP for small polyps.

¶Articles included in the network meta-analysis for 4–10-mm non-pedunculated polyps.

### Risk of bias assessment

Random sequence generation and allocation concealment were described in 91 and 96% of the studies, respectively, and 86% did not use selective reporting. No studies were blinded because it was impossible to blind the endoscopist. Because of the thermal effects of convenient polypectomy, the blinding of pathologists is unrealistic. Detection bias and attrition bias were varied in different outcomes (see Supplementary Figures 1A–E for risk of bias assessment).

### Meta-analysis and subgroup analysis compared HSP and CSP for polyps of <10 mm

**FIGURE 3:**
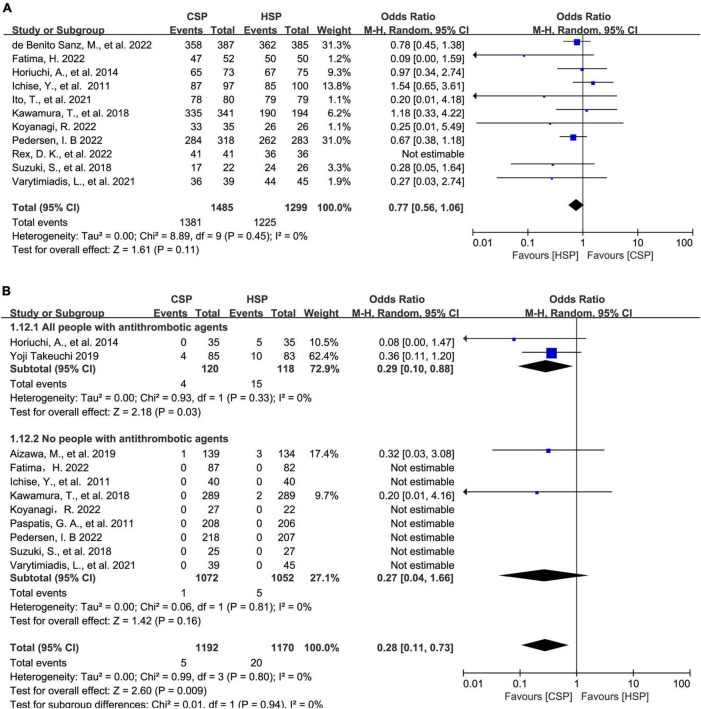
Direct meta-analysis and subgroup analysis comparing hot snare polypectomy (HSP) and cold snare polypectomy (CSP) for polyps of <10 mm. **(A)** Forest plot of complete resection rate. **(B)** Forest plot of major post-polypectomy bleeding rate: subgroups of patients on/not on antithrombotic agents.

A Total of 14 articles compared HSP versus CSP for polyps of <10 mm. For complete resection rate, CSP seemed to have a lower complete resection rate to that of HSP although with no statistically significant difference (OR: 0.77, 95% CI: 0.56–1.06, *p* = 0.11, *I*^2^ = 0%) (Figure 3A). CSP was similar to HSP for polyp retrieval rate (96.6% vs. 97.3%; OR: 0.78, 95% CI: 0.53–1.15, *P* = 0.20, *I*^2^ = 0%) (Supplementary Figure 2A). In subgroup analysis based on patients on antithrombotic agents, CSP had a lower rate of PPB in both groups, although with no statistically significant difference (Supplementary Figure 2B). Furthermore, for major PPB rate, CSP showed a lower risk of major PPB than HSP (OR: 0.28, 95% CI: 0.11–0.73, *P* = 0.009, *I*^2^ = 0%) and was same for people on antithrombotic drugs in subgroup analysis (3.0 vs. 12.7%, OR: 0.29, 95% CI: 0.10–0.88, *P* = 0.03, *I*^2^ = 0%) (Figure 3B). Three articles reported total colonoscopy time and four reported specific polypectomy time. These studies were analyzed separately. Polypectomy time was shorter for CSP (WMD: −0.42 min, 95% CI [−0.65, −0.19], *p* < 0.001). Total colonoscopy time showed a similar conclusion of shorter operating time in CSP versus HSP (WMD: −7.13 min, 95% CI [−8.94, −5.32], *p* < 0.001) (Supplementary Figures 2C, D).

### Pair-wise and network meta-analysis for small (4–10 mm) non-pedunculated polyps

#### Pair-wise meta-analysis for small (4–10 mm) non-pedunculated polyps

A total of 17 articles for treating small (4–10 mm) non-pedunculated polyps were included in the NMA. All patients were not on antithrombotic drugs in evaluating security. Complete resection rate, polyp retrieval rate, IB rate, PPB, procedure time of CSP and other resection methods derived from pair-wise meta-analysis are shown in Supplementary Figures 3A–E. For all pair-wise meta-analyses, HSP had a higher complete resection rate than CSP (OR: 3.1, 95% CI: 1.2–15, *I*^2^ = 48.9%). CSP had a significantly shorter time than HSP and EMR (WMD: −16.55 s, 95% CI [−25.25 s, −7.84 s], *p* < 0.001), (WMD: –48.00 s, 95% CI [−79.46, −16.54 s], *p* = 0.003). No statistically significant difference in other outcome between different treatments were found.

#### Network meta-analysis and sensitivity analysis for small (4–10 mm) non-pedunculated polyps and SUCRA value of treatments

**FIGURE 4:**
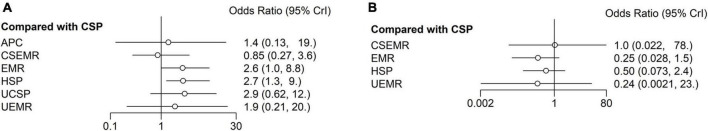
Forest plots reporting the results of the network meta-analysis. **(A)** Complete resection rate. **(B)** Intraprocedural bleeding rate. Reference was CSP. CSP, cold snare polypectomy; HSP, hot snare polypectomy; EMR, endoscopic mucosal resection; APC, argon plasma coagulation; UCSP, underwater cold snare polypectomy; CS-EMR, cold snare endoscopic mucosal resection; UEMR, underwater endoscopic mucosal resection.

For complete resection rate, 17 studies, including multi-arm studies, compared seven different treatments (4,361 polyps) (27–29, 31–44). Combining direct and indirect evidence, HSP and EMR had higher complete resection rates than CSP in the NMA (OR: 2.7, 95% CI: 1.3–9.2, *I*^2^ = 27.7%, with moderate quality), (OR: 2.6, 95% CI: 1.0–8.8, *I*^2^ = 55%, with low quality), (Figure 4A; Supplementary Table 2). For SUCRA score, HSP ranked first (0.71), followed by EMR (0.70), UCSP (0.70), UEMR (0.52), APC (0.43), CSP (0.23), and CS-EMR (0.20). It should be noted that there was only one article that evaluated APC and UEMR, respectively.

Sensitivity analysis for the primary outcome showed similar outcome. After excluding 4 articles using R0 resection rate (33, 35, 38, 42). In NMA of 13 articles, HSP had higher complete resection rate than CSP (OR: 2.7, 95% CI: 1.1–13, *I*^2^ = 33.1%) confirming the outcomes of the primary analyses (Supplementary Figure 4).

For polyp retrieval rate, six studies compared five different treatments (2,255 polyps) (26, 36, 38, 40, 42, 43). One study (42) reported UCSP with a 100% polyp retrieval rate, followed by HSP with a polyp retrieval rate of 99.5% (95% CI: 0.99, 1.00), EMR with pooled retrieval rate of 99.2% (95% CI: 0.99, 1.00). CSP ranked forth with a pooled retrieval rate of 97.9% (95% CI: 0.98, 0.99). CS-EMR ranked last with a pooled retrieval rate of 95.6% (95% CI: 0.93, 0.98) and there was no information about the UEMR group.

For IB rate, eleven articles compared five different treatments (2,670 polyps) (27, 32–38, 40, 43, 44). Besides two articles reported no IB in the UCSP group (0/279 polyps), (41, 42) and one three-arm study reported no IB in the APC group (0/39 polyps) (33). No statistically significant differences were found between the five different treatments (Figure 4B; Supplementary Table 3). For SUCRA score, EMR ranked first (0.78), followed by UEMR (0.65), HSP (0.50), CS-EMR (0.32), and CSP (0.25). All cases of IB were successfully treated by endoscopic hemostasis. No perforation occurred in any of the studies.

For major PPB and PPB, 14 articles compared seven treatments (3,316 patients) (27, 29, 30, 32–37, 39, 40, 42–44). Only three patients presented major PPB requiring medical intervention. The information can be delineated as follows: HSP (2/1148), EMR (1/586), CSP (0/1241), UCSP (0/56), CS-EMR (0/182), UEMR (0/66), and APC (0/37). Some articles did not report mild hematochezia. Six articles reported different degrees of hematochezia, (27, 29, 30, 32, 42, 44) and the rate of PPB range from 0 to 1.8%. No perforation occurred in any of the studies.

### Transitivity, consistency, and heterogeneity

For transitivity, our NMA only included 4–10 mm non-pedunculated polyps. No patient was on antithrombotic drugs when evaluating the post-polypectomy complications. Variables such as patient age, adenoma ratio, and average polyp size were similar in the NMA (Table 1). For assessment of consistency, the node-splitting model did not reveal any significant difference in the comparisons for all outcomes. Consistency test for primary outcome was shown in Supplementary Figure 5. In the heterogeneity analysis through Bayesian meta-analysis, the global *I*^2^ was 23.5 and 6.7% for complete resection rate and IB rate. The heterogeneity of pair-wise meta-analysis are shown in Supplementary Figures 3A, C.

### GRADE evaluation of quality of evidence

Grading of recommendations assessment, development, and evaluation indicated that evidence quality ranged from very low to moderate; in fact, it was rated low or moderate for most comparisons (Supplementary Table 4). All studies were at high risk of bias because of their design was unable to blind endoscopists, and many studies were imprecise due to confidence intervals across the invalid line. Therefore, the majority of studies were downgraded by one or two levels.

## Discussion

For the largest meta-analysis of 14 RCTs comparing HSP with CSP for polyps of <10 mm, we found HSP tended to be superior to CSP in completely resection rate although with no statistically significant difference and revealed lower major PPB rate in the CSP group compared to the HSP group, especially for patients on antithrombotic agents.

For 4–10 mm non-pedunculated polyps, after excluding diminutive polyps, HSP had a higher complete resection rate than CSP not only in NMA but also in pair-wise meta-analysis. EMR had a higher complete resection rate than CSP in NMA. UCSP shows great potential with high efficiency and safety. The rate of PPB ranged from 0 to 1.8% in patients not taking antithrombotic agents and only three patients presented major PPB requiring medical intervention (3/3,316), two patients in HSP group (2/1,148) and one in EMR group (1/586). All cases of IB and major PPB were successfully treated by endoscopic hemostasis.

We have demonstrated that CSP is safer than HSP, especially for patients on antithrombotic drugs, through evidence-based medical research. This could due to the fact that HSP leads to damage to the deep layer of the colon wall, involving more large blood vessels (15, 45). With the increased use of antithrombotic drugs, our study proved that CSP should be performed on patients taking antithrombotic drugs based on the guidelines (46).

As the primary outcome, the method to use to evaluate the complete resection rate remains controversial. Three methods were used for evaluating the complete resection rate in different RCTs. The most common method was random biopsies from the horizontal and vertical edges of the margins of the mucosal defect. However, only partial margins were evaluated, which would overestimate the complete resection rate. The second method was histologically evaluated negative lateral and vertical margins for neoplasia (R0 resection rate), but the margin of some specimens were unassessable, and may have been truly negative margins. Other studies defined “complete resection” as no recurrence in the resection site following surveillance colonoscopy, which was probably the best evaluation method for complete resection rate, but it was not feasible to detect post-polypectomy scars. Therefore, we were unable to accurately evaluate the value of complete resection rates.

The lower complete resection rate in the CSP group compared to the HSP and EMR for 4–10 mm non-pedunculated polyps. The reason may be multifold. First, several articles demonstrated that the resection depth of CSP was shallow and the submucosal layer was obtained less in the CSP group than in the HSP group (28, 32). Second, many articles did not report information regarding extended excision in the CSP group. CSP may resect specimens without sufficiently clear margins, but securing at least a 2 mm clear margin of normal tissue is important in CSP so that eradication of neoplastic tissue can be assured (47). Third, electrocautery of HSP and EMR can eliminate the possible residual polyp. Finally, we encountered different methods to evaluate the complete resection rate. Some articles pointed out that specimens from CSP or HSP are not suitable for histological evaluation due to the tissue damage caused during the retrieval process through the working channel and higher Rx resection rate (the involvement of the resection margin could not be determined), especially in cold snare resection (48, 49). Although it is still unclear whether Rx would be an independent risk factor for polyp recurrence, Rx resection of specimens can be equally detrimental to patients as early repeat colonoscopy is still needed (17). And after excluding articles using R0 resection rate, sensitivity analysis showed similar outcome to our primary analysis. Meanwhile, as the most accurate evaluation method, recent RCTs also found higher rates of polyp recurrence in the CSP group in follow-up colonoscopy, although with no statistically significant difference (28, 33, 35). More high-quality evidence on findings at surveillance colonoscopy is needed.

Underwater cold snare polypectomy has emerged as an viable alternative to conventional CSP (50). UCSP performed better than CSP in our NMA, which is consistent with the findings of a previous propensity score-matching study (50). For two RCTs and one propensity score-matching study (281 patients), no IB or PPB was reported, and the specimen retrieval rate was 100% (41, 42). Underwater polypectomy was first described by Binmoeller for resecting large colorectal lesions (51). In the UCSP group, snaring of the polyp with adequate normal mucosa around the lesion was relatively easy, and the rate of resection with muscularis mucosa in the UCSP group was significantly higher than that in the CSP group (50). Water immersion improves the visibility and operability of the endoscope. By sucking the specimen and water at the same time, it is possible to retrieve the resected specimen more easily and quickly (42). UCSP shows great potential for resecting 4–10 mm non-pedunculated polyps; however, the number of studies of UCSP is limited, and new RCT has designed (52).

Our study had some limitations. First, we did not distinguish traditional cold snare and dedicated cold snare. Studies have shown that the effect of the dedicated cold snare is better than that of the traditional cold snare (53, 54). However, the type of snare was freely chosen in some RCTs. Secondly, all studies were at high risk of bias due to their design and were inevitably. Finally, our study did not perform a comparative cost-effectiveness analysis, which is also important to consider when evaluating resection methods, but the evidence is relatively sparse.

Despite the above limitations, we believe that our study has unique strengths. First, we performed NMA for small polyps, especially for 4–10 mm polyps which was comprehensive and rigorous but rarely used in comparing different methods for polyp treatments. Second, for polyps <10 mm, the largest meta-analysis and subgroup analysis was conducted to compare the efficiency and safety of CSP versus HSP, complementing and updating other evidence-based medical research.

Through evidence-based medicine we found that CSP is safer than HSP for polyps <10 mm. What’s more, for 4–10 mm non-pedunculated polyps, EMR and HSP has an advantage over CSP in improving complete resection rates, and could be relatively safely used in patients who were not taking antithrombotic agents. Finally, UCSP as a new polyp removal method deserved further study.

## Data availability statement

The original contributions presented in this study are included in the article/Supplementary material, further inquiries can be directed to the corresponding author.

## Author contributions

XL and HX: conception and design. XL, HZ, RL, and HX: analysis and interpretation of data. XL, HZ, and FL: drafting of the manuscript. HX: critical revision of the manuscript for important intellectual content. XL, HZ, FL, RL, and HX: final approval of the manuscript. All authors contributed to the article and approved the submitted version.
